# Non‐psychotropic *Cannabis sativa* L. phytocomplex modulates microglial inflammatory response through CB2 receptors‐, endocannabinoids‐, and NF‐κB‐mediated signaling

**DOI:** 10.1002/ptr.7458

**Published:** 2022-04-08

**Authors:** Vittoria Borgonetti, Cristina Benatti, Paolo Governa, Giovanni Isoldi, Federica Pellati, Silvia Alboni, Fabio Tascedda, Monica Montopoli, Nicoletta Galeotti, Fabrizio Manetti, Elisabetta Miraldi, Marco Biagi, Giovanna Rigillo

**Affiliations:** ^1^ Department of Neuroscience, Psychology, Drug Research and Child Health (NEUROFARBA), Section of Pharmacology University of Florence Florence Italy; ^2^ Center for Neuroscience and Neurotechnology University of Modena and Reggio Emilia Modena Italy; ^3^ Department of Life Sciences University of Modena and Reggio Emilia Modena Italy; ^4^ Department of Biotechnology, Chemistry and Pharmacy (Department of Excellence 2018‐2022) University of Siena Siena Italy; ^5^ Materia Medica Processing S.r.l. Siena Italy; ^6^ Consorzio Interuniversitario Biotecnologie Trieste Italy; ^7^ Department of Pharmaceutical and Pharmacological Sciences University of Padua Padua Italy; ^8^ Department of Physical Sciences, Earth and Environment University of Siena Siena Italy

**Keywords:** cannabidiol, *Cannabis sativa* L., inflammation, microglia, phytocomplex, β‐Caryophyllene

## Abstract

*Cannabis sativa* L. is increasingly emerging for its protective role in modulating neuroinflammation, a complex process orchestrated among others by microglia, the resident immune cells of the central nervous system. Phytocannabinoids, especially cannabidiol (CBD), terpenes, and other constituents trigger several upstream and downstream microglial intracellular pathways. Here, we investigated the molecular mechanisms of a CBD‐ and terpenes‐enriched *C*. *sativa* extract (CSE) in an in vitro model of neuroinflammation. We evaluated the effect of CSE on the inflammatory response induced by exposure to lipopolysaccharide (LPS) in BV‐2 microglial cells, compared with CBD and β‐caryophyllene (CAR), CB2 receptors (CB2r) inverse and full agonist, respectively. The LPS‐induced upregulation of the pro‐inflammatory cytokines IL‐1β, IL‐6, and TNF‐α was significantly attenuated by CSE and only partially by CBD, whereas CAR was ineffective. In BV‐2 cells, these anti‐inflammatory effects exerted by CSE phytocomplex were only partially dependent on CB2r modulation and they were mediated by the regulation of enzymes responsible for the endocannabinoids metabolism, by the inhibition of reactive oxygen species release and the modulation of JNK/p38 cascade with consequent NF‐κB p65 nuclear translocation suppression. Our data suggest that *C*. *sativa* phytocomplex and its multitarget mechanism could represent a novel therapeutic strategy for neuroinflammatory‐related diseases.

## 1 INTRODUCTION

Sound scientific evidence reports that the activation of the endocannabinoid system (ECS) is crucial in the inflammatory process modulation: the stimulation of cannabinoid receptors (CBrs) by agonists and inverse agonists leads to the activation of several intracellular pathways counteracting the inflammatory cascade (McKenna & McDougall, 2020; Oláh, Szekanecz, & Bíró, 2017). Although both CB1 and CB2 receptors have been detected in the immune system cells, CB2 receptors (CB2r) have a more specific pattern of expression in these cells, possessing a crucial role in cannabinoid system‐mediated regulation of inflammatory and immunity processes (Atwood & MacKie, 2010). In the central nervous system (CNS), the majority of CB1 receptors (CB1r)‐expressing cells are neurons while CB2r are marginally expressed in the healthy brain and undergo a modulation that depends on the activation state of microglia (Carlisle, Marciano‐Cabral, Staab, Ludwick, & Cabral, 2002; Maresz, Carrier, Ponomarev, Hillard, & Dittel, 2005; Mecha, Carrillo‐Salinas, Feliú, Mestre, & Guaza, 2016; Stella, 2010). This selective microglial expression points at considering CB2r modulation particularly relevant in neuro‐disorders (Kendall & Yudowski, 2017).

CBrs are primarily activated by endogenous cannabinoids, anandamide (AEA) and 2‐arachidonoylglycerol (2‐AG), but also by exogenous ligands, such as phytocannabinoids. The endogenous cannabinoids are considered for their neuroprotective properties in neuroinflammatory‐related disorders: interventions on the endocannabinoid signaling or on the metabolism of AEA and 2‐AG have demonstrated an important role of ECS in maintaining the integrity of the brain and controlling inflammatory response in neuropathology (Alhouayek, Masquelier, & Muccioli, 2014; Di Marzo et al., 1994; Ruhl, Corsten, Beier, & Kim, 2020; Scotter, Abood, & Glass, 2010; Xu & Chen, 2015).

*Cannabis sativa* L. and its CBrs ligands have gained a prominent role in the field of neuroinflammation. In this context, the effect of treatment with the psychoactive Δ^9^‐tetrahydrocannabinol (THC) and its synthetic derivatives has been investigated in several neurodegenerative diseases (Herrmann et al., 2019; Scotter et al., 2010), but the controversial balance between symptoms relief, life quality, and side effects is still debated (Afrin et al., 2020). On the other hand, cannabidiol (CBD), the major non‐psychotropic constituent of *C*. *sativa*, has been studied in numerous human inflammatory conditions and oxidative stress related‐diseases within the CNS (Pellati, Borgonetti, et al., 2018; Pellati, Brighenti, et al., 2018), such as Parkinson's and Alzheimer's diseases (Cassano et al., 2020; Esposito et al., 2011). Among the proposed mechanisms underlying CBD activity has been reported its ability to interfere with the endogenous AEA metabolism increasing the AEA‐mediated anti‐inflammatory and neuroprotective effects (Bisogno et al., 2001; Leweke et al., 2012).

Given its excellent safety and tolerability profile exhibited in clinical studies, CBD has a high potential as a therapeutic agent for the treatment of neurodegenerative disorders, through a multifaceted mechanism of action, both CB receptor‐dependent and ‐independent (Borgonetti, Governa, Montopoli, & Biagi, 2019). Interestingly, *C*. *sativa* cannabinoids and non‐cannabinoid constituents have been shown to exert anti‐inflammatory activity in CNS by modulating the main pathway involved in microglial response: the nuclear factor kappa B (NF‐κB), the extracellular signal‐regulated kinases (ERK), p38 and c‐Jun N‐terminal kinases (JNK) mitogen‐activated protein kinases (MAPKs) (Borgonetti et al., 2019; Karin & Delhase, 2000; Raingeaud et al., 1995; Shu et al., 2014; Xiang, Xiao, Shen, & Li, 2018). In rat microglial cells stimulated with micromolar concentrations of the endotoxin lipopolysaccharide (LPS), a known inflammation activator, CBD inhibited NF‐κB translocation (Kozela et al., 2010), while exposure of human glioma cells to CBD caused a concentration‐dependent downregulation of ERK and Akt signaling pathways (Solinas et al., 2013). Besides terpenphenolic cannabinoids, β‐caryophyllene (CAR), the most important component of cannabis terpene fraction, as well as a full agonist of CB2r, showed a protective effect against neuroinflammation both in vitro and in vivo models (Lindsey et al., 2019). In LPS‐induced microglial M1/M2 imbalance, CAR exerted a protective effect providing the release of the anti‐inflammatory molecules and decreasing inflammatory and oxidative biomarkers (Askari & Shafiee‐Nick, 2019). At nanomolar concentrations, CAR inhibited IL‐1β and TNF‐α expression, as well as ERK phosphorylation, in LPS‐stimulated human peripheral blood mononuclear cells (PBMC) (Gertsch et al., 2008). Other minor phytocannabinoids, such as cannabigerol (CBG) and its derivates, have shown promising neuroprotective potential in neurodegenerative diseases (García et al., 2018; Stone, Murphy, England, & O'Sullivan, 2020). Flavonoids represent a minor phytochemical class of *C*. *sativa* components, but compounds such as apigenin and luteolin, which have a clear anti‐inflammatory profile, occur in the phytocomplex of the species (Borgonetti et al., 2019; Che et al., 2020; Chumsakul et al., 2020; Kao et al., 2011).

Despite the extensive literature available on THC, CBD, and other individual constituents of *C*. *sativa*, the pharmacological effects of the whole cannabis phytocomplex in microglial inflammatory stress are still poorly investigated. This scientific gap has actually slowed down the use of medical cannabis, as phytocomplex, in neuroinflammatory diseases and much more the use of cannabis with low THC, although past and current evidence highlights the beneficial effects of employing the full extracts over single compounds, such as in spasticity (Vermersch & Trojano, 2016) or pain relief (Capano, Weaver, & Burkman, 2020). These reasons led us to elucidate the anti‐inflammatory potential of a standardized extract of non‐psychotropic *C*. *sativa* in an in vitro model of LPS‐induced microglial inflammation compared to those of CBD and CAR.

## 2 MATERIALS AND METHODS

### 2.1 *C*. *sativa* L. extraction and chemicals

A standardized *C*. *sativa* L. extract (CSE) (Carmagnola variety), obtained from the dried inflorescence, was prepared by the research laboratory of the Italian Society of Phytotherapy (SIFITLab) using an automatic extractor Tecnolab TIMATIC (Spello, Perugia, Italy) with supercritical gas solvent. The herbal material was previously decarboxylated in an oven at 120 °C for 1 hr and then extracted for 5 hr at 35 °C, operating at 7 bars. CBD and CAR (Merck KGaA, Germany) were used as standard references for each experiment.

### 2.2 Chemical analysis

#### 2.2.1 HPLC–diode‐array detection analysis of cannabinoids and flavonoids

CSE was analyzed by HPLC–diode‐array detection (DAD) using a Shimadzu Prominence LC 2030 3D instrument equipped with a Bondapak® C18 column (300 × 3.9 mm, 10 μm) (Waters Corporation, MA). Water +0.1% vol/vol formic acid (A) and methanol +0.1% vol/vol formic acid (B) were used as the mobile phase according to the methods described as follows. Cannabinoids: A 35% at 0 min for 3 min, then from 35 to 10% after 10 min, A 10% for 2 min, then from 10 to 35% at 14 min, and finally A 35% for 1 min; flow rate was set at 1.2 ml/min. Chromatograms were recorded at 225 nm. Flavonoids: A from 90 to 75% at 0 min to 75% at 15 min, 65% at 18 min, and finally 50% at 25 min; flow rate was set at 0.8 ml/min. Chromatograms were recorded at 350 nm. Analyses were performed using 10 μl of CSE solution in ethanol 96% vol/vol (10 mg/ml) and CBD, CAR, vitexin, and apigenin were used as external standards (purity >99%) (Merck KGaA, Germany). The correlation coefficient (*R* ^2^) of each curve was >0.99. CBD acid (CBDA), THC, its acidic precursor (THCA), and other phytocannabinoids were quantified according to response factors related to CBD published in Analytical Monograph Cannabis Flos version 7.1 released by Cannabis Bureau, Netherland (OMC/Farmalyse BV, 2014).

#### 2.2.2 GC–MS analysis of volatile compounds

Analyses were performed on a 7820A gas chromatograph coupled with a 5975C network mass spectrometer (GC–MS) (Agilent Technologies, Germany). Compounds were separated on an Agilent Technologies HP‐5 MS cross‐linked poly‐5% diphenyl–95% dimethyl polysiloxane (30 m × 0.32 mm i.d., 0.25 μm film thickness) capillary column. The column temperature was initially set at 45 °C, then increased at a rate of 2 °C/min up to 100 °C, then raised to 250 °C at a rate of 5°C/min and again raised up to 280 °C at a rate of 11 °C/min and finally held for 15 min. The injection volume was 0.1 μl, with a split ratio of 1:40. Helium was used as the carrier gas, at a flow rate of 0.7 ml/min. The injector, transfer line, and ion‐source temperature were 250, 280, and 230 °C, respectively. MS detection was performed with electron ionization at 70 eV, operating in the full‐scan acquisition mode in the *m*/*z* range 40–400. The sample was diluted 1:20 (vol/vol) with *n*‐hexane before GC–MS analysis. All reference standards used for GC analysis, chromatographic grade organic solvents and reagents were purchased from Merck KGaA (Germany).

#### 2.2.3 GC–FID analysis of volatile compounds

Analyses were carried out on a GC coupled with a flame ionization detector (FID) Agilent Technologies 7820A. Compounds were separated on an Agilent Technologies HP‐5 cross‐linked poly‐5% diphenyl–95% dimethyl polysiloxane (30 m × 0.32 mm i.d., 0.25 μm film thickness) capillary column. The temperature program was the same as described above. The injection volume was 0.1 μl in the split mode 1:20. Helium was used as the carrier gas at a flow rate of 1.0 ml/min. The injector and detector temperature were set at 250 and 300°C, respectively. The sample and the reference standards were diluted 1:20 (vol/vol) with *n*‐hexane before GC–FID analysis. The analyses were performed in triplicate.

#### 2.2.4 Qualitative and semi‐quantitative analysis of volatile compounds

The compounds in the sample analyzed were identified by comparing the retention times of the chromatographic peaks with those of authentic reference standards run under the same conditions and by comparing the experimental linear retention index values, calculated from a mixture of *n*‐alkanes (C_8_–C_40_) in *n*‐hexane and injected under the same conditions, with those previously described in the literature. Peak enrichment by co‐injection with authentic reference compounds was also carried out. A comparison of the MS‐fragmentation pattern of the target analytes with those of pure components was performed. A mass‐spectrum database search was performed using the National Institute of Standards and Technology mass‐spectral database (version 1.4). The relative amounts of each component were expressed as percent peak area relative to the total peak area.

### 2.3 Cell culture and treatments

The murine microglial cells BV‐2 were provided by the Department of Life Science (University of Modena and Reggio Emilia). Cells were cultured at 37 °C and 5% CO_2_ in RPMI 1640 (Merck KGaA, Germany) supplemented with 10% heat‐inactivated fetal bovine serum (FBS) (Merck KGaA, Germany), 1% penicillin/streptomycin solution (Merck KGaA, Germany), and 1% of l‐glutammine (Merck KGaA, Germany) and passed by trypsinization as previously described (Borgonetti, Governa, Biagi, Dalia, & Corsi, 2020). Cells under passage 20 were used for all experiments. For the LPS time course: cells were harvested 2, 6, or 24 hr after treatment with 250 ng/ml of bacterial LPS (from Gram‐negative *Salmonella enteritidis*, #L7770, Merck KGaA), while the control group received phosphate‐buffered saline (PBS). BV‐2 cells were pre‐treated with CSE, CBD, or CAR (1 μg/ml) for 4 hr as reported in each of the following sections, then stimulated with LPS (250 ng/ml). All treatments were performed in low serum‐supplemented medium (3% FBS). Cells were collected at times indicated in each section for further analysis.

### 2.4 Cytokines dosage

BV‐2 cells (1 × 10^5^ cells/well) were seeded into 24‐well plates and cultured for 24 hr. Cells were pre‐treated for 4 hr with CSE, CBD, or CAR (1 μg/ml) and then stimulated with LPS (250 ng/ml) for 2 hr. TNF‐α, IL‐6, and IL‐1β production were evaluated in BV‐2 cell lysate and medium together according to the manufacturer's instruction by non‐competitive sandwich ELISA (Biolegend e‐Bioscience DX Diagnostic, CA) dosages, as previously reported (Governa et al., 2019). Absorbance was measured at 450 nm using an MP96 microplate reader spectrophotometer (Safas, Montecarlo). Samples were assayed in duplicate. Dosages were performed in three independent experiments.

### 2.5 Total RNA extraction, reverse transcription, and real‐time PCR

BV‐2 cells were seeded in 6‐well plates at the density of 2 × 10^6^ cells/well and cultured for 24 hr. Cells were pre‐treated either with CSE, CBD, or CAR (1 μg/ml) then stimulated with LPS (250 ng/ml) for 2 hr. RNA extraction and DNAse treatment were performed as previously described (Rigillo et al., 2018) using GenElute™ Mammalian Total RNA Miniprep Kit and DNASE70‐On‐Column DNase I Digestion Set (Merck KGaA, Germany). Two micrograms of total RNA were reverse transcribed with High Capacity cDNA Reverse Transcription Kit (Thermo Fisher Scientific, MA) and RT‐qPCR was performed in CFX Connect Real‐Time PCR machine (Bio‐Rad Laboratories, CA), using SsoAdvanced Universal SYBR Green Supermix (Bio‐Rad Laboratories, CA) and specific forward and reverse primers at a final concentration of 300 nM (Table 1) as previously described (Caruso et al., 2021). The optimized cycling conditions were as follows: initial denaturation at 95 °C for 30 s, followed by 40 cycles of denaturation at 95 °C for 10 s, primer annealing at 60 °C for 30 s. PCR products were subjected to a melting curve analysis (a gradual increase of temperature from 60 to 95 °C in increments of 0.5 °C with continuous reading of fluorescence) and agarose gel separation to verify the absence of artifacts, such as primer‐dimers or non‐specific products. Cycle threshold (Cq) value was determined by the CFX maestro software (Bio‐Rad Laboratories, CA), mRNA expression was calculated with the ΔΔCt method with glyceraldehydes‐3‐phosphate dehydrogenase (GAPDH) as endogenous control. For gene expression analysis, endogenous control mRNA levels were not affected among treatments (*p* > .05, one‐way analysis of variance [ANOVA]).

**TABLE 1:** Transcript and sequence of each primer used in real‐time PCR

Transcript	NCBI GenBank	Primer sequence
*Mus musculus* cannabinoid receptor 1 (*CB1r*)	*NM_007726*.*3*	Fw CTGGTTCTGATCCTGGTGGT Rv TGTCTCAGGTCCTTGCTCCT
*Mus musculus* cannabinoid receptor 2 (*CB2r*)	*NM_009924*.*4*	Fw TCATTGCCATCCTCTTTTCC Rv GAACCAGCATATGAGCAGCA
Glyceraldehyde 3‐phosphate dehydrogenase *(GAPDH)*	*NM_017008*.*3*	Fw AAGGTCATCCATGACAACTTTG Rv GGGCCATCCACAGTCTTCTG
*Mus Musculus* diacylglycerol lipase *β (DAGLβ)*	*NM_144915*	Fw CTTCTCCACCAGCAACAAGA Rv AGTTCTCCACTTCTGCATCTAAC
*Mus Musculus* fatty acid amide hydrolase *(FAAH)*	*NM_010173*	Fw ACTGGACTGAGGAAGGACTATG Rv GGAGACTTATTTGGCTGGGAAG
*Mus Musculus N*‐acyl phosphatidylethanolamine phospholipase d *(NAPE‐PLD)*	*NM_178728*	Fw CAGACTAGAGGAGGACGTAACT Rv TCAGCCATCTGAGCACATTC

### 2.6 Protein extraction and western blotting

BV‐2 cells were seeded in 6‐well plates at the density of 1 × 10^6^ cells/well and cultured for 24 hr. Cells were pre‐treated either with CSE, CBD, or CAR (1 μg/ml) then stimulated with LPS (250 ng/ml) for 2 hr. Whole‐cell protein extracts were prepared by resuspending cells into 1× sodium dodecyl sulfate (SDS) sample buffer (25 mM Tris–HCl pH 6.8, 1.5 mM EDTA, 20% glycerol, 2% SDS, 5% β‐mercaptoethanol, and 0.0025% bromophenol blue). The protein concentration of the extracts was determined using the standard protocol Coomassie reagent (Thermo Fisher Scientific, MA). For immunoblotting equivalent amounts of extracts were resolved by SDS–polyacrylamide gel electrophoresis (PAGE), electrotransferred to polyvinylidene fluoride membrane (Millipore, MA), as previously described (Benatti et al., 2018). Membranes were incubated with primary antibodies: anti‐CB2r (1:1,000, sc‐25494, Santa Cruz Biotechnology, Texas), anti‐Vinculin (1:10,000, #V4504, Merck KGaA, Germany) in blocking‐buffer. Secondary antibody: anti‐rabbit IgG‐HRP‐linked (1:5,000, #7071, Cell Signaling, MA) for all targets. Bands were detected using Immobilon Western Chemiluminescent HRP (Millipore, MA). The levels of protein were calculated by measuring the peak densitometric area of the autoradiography analysed with an image analyser (GS‐690, Bio‐Rad Laboratories, CA). Each experiment was performed three times and the mean of the optical densities (OD) ratios (target/internal standard) was analysed. The OD of CB2r signals were normalized according to the OD of vinculin. Ratios were expressed as a percentage of untreated BV‐2 cells ± standard error of the mean (SEM).

### 2.7 MAPKs activation

MAPKs (ERK1/2, JNK, and p38) activation was evaluated using non‐competitive sandwich ELISA (Biolegend e‐Bioscience DX Diagnostic, MA), according to the manufacturer's instructions. BV‐2 cells (1 × 10^5^ cells/well) were seeded into 24‐well plates and cultured for 24 hr. Cells were pre‐treated for 4 hr with CSE (1 μg/ml) then stimulated with LPS (250 ng/ml) for 30, 60, and 120 min, and lysed with 100 μl of the provided lysis buffer. The total protein content of each sample was evaluated by Bradford colorimetric method (Merck KGaA; Germany), using bovine serum albumi (BSA) (Merck KGaA, Germany) as a reference standard. Absorbance was measured at 450 nm using an MP96 microplate reader spectrophotometer (Safas, Monaco). The activation of MAPKs was calculated as the ratio of phosphorylated to total proteins, normalizing values to the untreated control. Three independent experiments were performed.

### 2.8 NF‐κB nuclear translocation

The nuclear translocation of the NF‐κB p65 subunit was evaluated by non‐competitive sandwich ELISA (Abcam, UK). BV‐2 cells (1 × 10^5^ cells/well) were seeded into 24‐well plates and grown to confluence. The medium was then replaced with fresh RPMI containing CSE treatment (1 μg/ml) for 4 hr before incubation with LPS (250 ng/ml) for 60 min as previously described (Borgonetti et al., 2020). Then, cells were washed three times with PBS and subcellular fractionation was obtained by applying lysis buffers with increasing detergent strength, as previously reported (Baghirova, Hughes, Hendzel, & Schulz, 2015). The total protein content of each sample was evaluated by Bradford colorimetric method (Merck KGaA, Germany), using BSA (Merck KGaA, Germany) as a reference standard. A non‐competitive sandwich ELISA assay was performed on both cytosol and nuclear cell lysates according to the supplier instructions. Absorbance was measured at 450 nm using an MP96 microplate reader spectrophotometer (Safas, Monaco). NF‐κB p65 nuclear translocation was calculated as the ratio of nuclear to total p65 subunit levels, normalizing values to the control. Three independent experiments were performed. Subsequently, nuclear translocation of phospho‐NF‐κB p65 was investigated by immunofluorescence. Briefly, 3 × 10^5^ cells were seeded on Superfrost® Plus Microscope slides (#041300, Menzel‐Glaser, Germany) and grown for 24 hr. BV‐2 cells were pre‐treated with CSE (1 μg/ml) for 4 hr then stimulated with LPS (250 ng/ml) for 60 min. After, cells were fixed with 4% paraformaldehyde for 15 min at room temperature (RT). Following incubation with blocking buffer (PBS, containing 1% BSA) for 1 hr at RT, primary anti‐phospho(S536)‐NF‐κB p65 antibody (1:200 in PBSA 5%; sc‐136548, Santa Cruz Biotechnology, MA) was added and incubated for 2 hr at RT. Cells were washed and incubated with secondary antibodies labeled with Invitrogen Alexa Fluor 568 (578‐603, 1:400; Thermo Fisher Scientific, MA) for 1 hr at RT. Slides were coverslipped using UltraCruz® Aqueous Mounting Medium with DAPI (Santa Cruz Biotechnology, MA) to identify the nucleus. Images were acquired with a Leica DM6000B fluorescence microscope. The immunofluorescence intensity was calculated by Image J (NIH).

### 2.9 Dosage of intracellular ROS species level

Reactive oxygen (ROS) production was quantified using 2′,7′‐dichlorodihydrofluorescein diacetate (H_2_‐DCF‐DA, Merck KGaA, Germany). Briefly, BV‐2 cells were seeded in 96‐well plates at a density of 5 × 10^3^ cells/well and cultured for 24 hr. Cells were treated with CSE (1 μg/ml) for 4 hr and stimulated with LPS (250 ng/ml) for further 2 hr, then incubated with a 50 μM H_2_DCF‐DA solution for 45 min at 37°C. In the presence of ROS, the reagent H_2_DCF‐DA was converted into a fluorescent adduct, dichlorofluorescein (DCF). DCF fluorescence intensity was measured at excitation 485 nm—emission 535 nm, using a Multilabel Plate Reader VICTOR X3 (PerkinElmer, MA). Three independent replicates were performed.

### 2.10 Antiradical capacity: DPPH assay

The antiradical capacity of CSE was tested through 2,2‐diphenyl‐1‐picrylhydrazyl (DPPH) assay, performing the method previously optimized by Biagi et al. (2019). The method is based on the absorbance measurement (515 nm) of the non‐radical form of DPPH (namely DPPH‐H) that is yielded by the reduction reaction of an alcoholic solution of DPPH in the presence of a hydrogen‐donating antioxidant. Tested samples were dissolved in ethanol 96% vol/vol. Different sample concentrations (range 6.25–0.19 mg/ml) were mixed with DPPH (1:19) and, after incubation for 30 min at RT in the dark, the absorbance was read at 515 nm using an MP96 microplate reader spectrophotometer (Safas, Monaco). IC_50_ values were calculated using linear regression analysis. Three independent replicates were performed.

### 2.11 Statistical analysis

Statistical analyses were performed using one‐way ANOVA followed by Tukey post‐hoc test (with *p* < .05 significance level). Data are presented as mean ± SEM. Analyses were conducted using SPSS for Windows® v.25 (SPSS Inc., IL) and Graphpad Prism (San Diego, CA).

### 2.12 Statement

In this study, requirements considered to be relevant in recent guidelines for best practice in natural products pharmacological research have been taken into account (Heinrich et al., 2018; Izzo et al., 2020).

## 3 RESULTS

### 3.1 Chemical analysis

The HPLC–DAD analysis revealed that CSE was highly rich in CBD, which represents the main single constituent of the extract (21.0% wt/wt). The decarboxylation of the plant material and the extraction procedure generated almost exclusively CBD in its neutral form, while its acidic precursor CBDA was <0.3% wt/wt.

**FIGURE 1:**
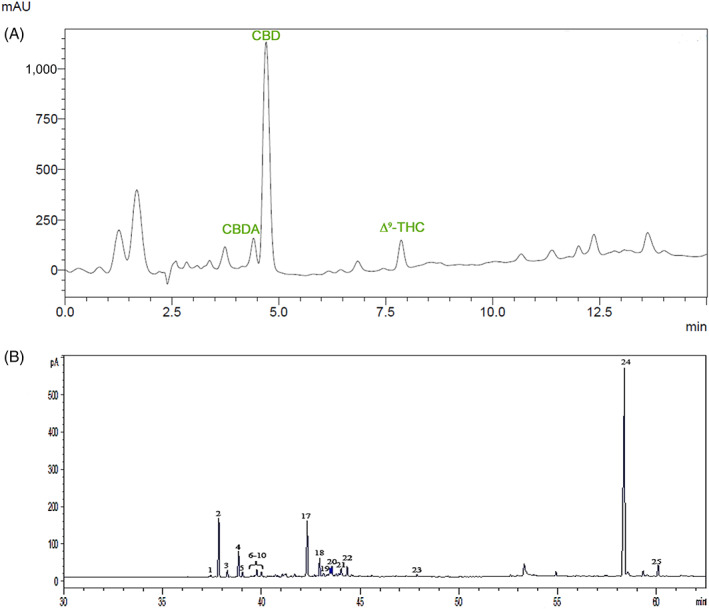
(A) Representative HPLC chromatogram of the *Cannabis sativa* L. extract recorded at 225 nm, following the method used for the analysis of cannabinoids. CBD, which elutes at 4.4 min, represents the main component. CBDA elutes at 4.2 min. The peak assigned to THC is at 7.4 min. (B) Representative GC chromatogram of the *C*. *sativa* L. extract, focused on the retention window of sesquiterpenes and cannabinoids. CBD (peak 24), CAR (peaks 2 and 17), and CBG (peak 25). For peak numbering, see Table S1. CAR, β‐caryophyllene; CBD, cannabidiol; CBDA, Cannabidiolic acid; CBG, Cannabigerol; GC, gas chromatography; THC, Δ^9^‐tetrahydrocannabinol

The raw extract was purified on a flash chromatography column to obtain a THC content <0.5% and CBG 1.0% wt/wt. The HPLC–DAD chromatogram recorded at 225 nm is shown in Figure 1a. The analysis of the flavonoid fraction by HPLC highlighted that this class of polyphenols was poorly represented in the extract and the sum of apigenin and vitexin was below 0.1% wt/wt. Moreover, the cannaflavins were investigated by comparison of the UV spectra of CSE analytes with published literature (Pellati, Borgonetti, et al., 2018; Pellati, Brighenti, et al., 2018) and they were not detected. The volatile fraction of CSE was analyzed using GC–MS and GC–FID; a representative chromatogram is shown in Figure 1b. Total terpenes content was 12.8% wt/wt of CSE. As expected, using this extraction procedure, monoterpenes were completely lost and only sesquiterpenes could be detected, with CAR, caryophyllene oxide and α‐humulene as the main constituents (3.2, 3.1, and 1.3% wt/wt respectively) (Table 2). The complete list of the 20 terpenes overall were detected in CSE volatile fraction is shown in the Table S1.

**TABLE 2:** Chemical characterization of CSE constituents by HPLC and GC analysis

Phytochemical profile		%wt/wt ± *SD*
Cannabinoids	Cannabidiol (CBD)	21.0% ± 0.6
	Cannabigerol (CBG)	1.0% ± 0.1
	Cannabidiolic acid (CBDA)	<0.3%
	THC + THCA	<0.5%
Terpenes	Total	12.8% ± 2.9
Sesquiterpenes	β‐caryophyllene (CAR) Caryophyllene‐oxide α‐Humulene	3.2% ± 0.8 3.1% ± 0.7 1.3% ± 0.3
Flavonoids	Apigenin	<0.1%

*Note*: Values are expressed as % wt/wt of extract weight.

Abbreviations: CSE, *Cannabis sativa* extract; GC, gas chromatography; THC, Δ^9^‐tetrahydrocannabinol.

### 3.2 Effect of LPS stimulation on pro‐inflammatory cytokines TNF‐α, IL‐6, and IL‐1β levels and CB2r expression in BV‐2 cells

**FIGURE 2:**
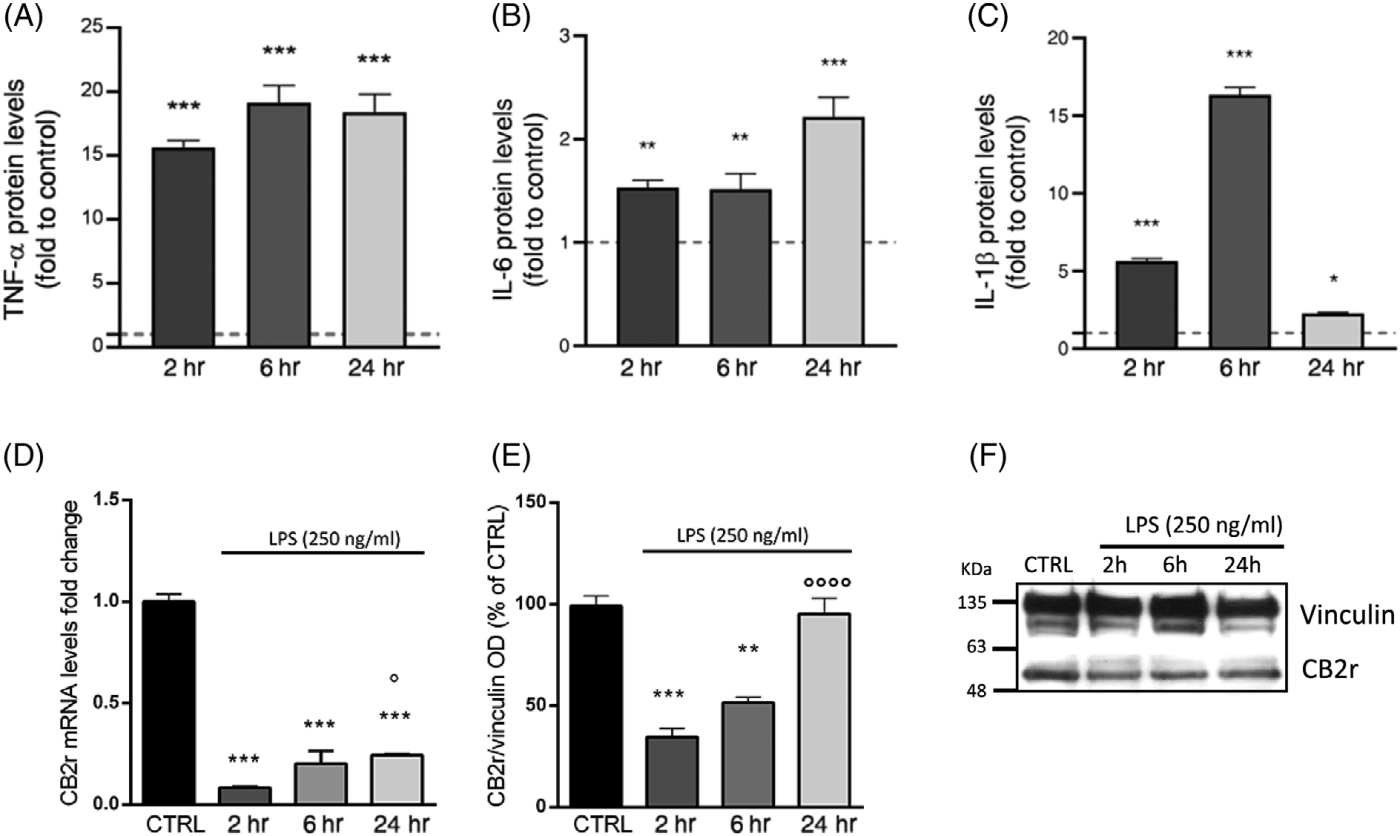
Protein expression of the pro‐inflammatory cytokines TNF‐α (A), IL‐6 (B), and IL‐1β (C) measured by ELISA assay in BV‐2 cells after a 2, 6, and 24 hr LPS stimulation (250 ng/ml). The dashed line represents the CTRL value set to 1 (*n* = 6). RT‐qPCR analysis of CB2r mRNA (D) and protein (E) levels measured by western blot in LPS‐time course treatment (2, 6, and 24 hr) in BV‐2 cells. (F) Immunoblot represents protein levels of CB2r and the endogenous target vinculin. Each column represents mean ± SEM. Data were analyzed by one‐way analysis of variance followed by Tukey: *p < .05, **p < .01,***p < .001 versus CTRL group; °*p* < .05, °°°°*p* < .0001 versus LPS (*n* = 5). LPS, lipopolysaccharide; SEM, standard error of the mean

To ensure the effectiveness of LPS stimulus on our microglia in vitro model, an ELISA assay was first used to measure the protein levels of the pro‐inflammatory cytokines TNF‐α, IL‐6, and IL‐1β in BV‐2 cells after 2, 6, and 24 hr of exposure to LPS. After 2 hr of LPS stimulation, TNF‐α resulted in the most upregulated cytokine with a significant increase of its levels by 15.62‐fold compared to the control group (Figure 2a). TNF‐α protein levels remained significantly higher than controls after 6 and 24 hr (19.00‐ and 18.40‐fold respectively; *F*(3;8) = 209.0; *p* < .001; Figure 2a). IL‐6 protein showed a different time course after the LPS exposure with an about 1.50‐fold increase at 2 and 6 hr, which was further enhanced to 2.21‐fold after 24 hr with respect to control (*F*(3;8) = 45.14; *p* < .001; Figure 2b). IL‐1β levels were significantly increased after a 2 hr exposure to LPS (5.62‐fold vs. CTRL) and continued to increase after 6 hr when the levels were 15.35‐fold higher than control cells. At 24 hr, IL‐1β levels of the group exposed to LPS were decreased with respect to the other time points, while remaining higher than the control group (1.25‐fold; *F*(3;8) = 21.36; *p* < .05; Figure 2c). Next, we investigated CBrs expression regulation in the microglial inflammatory response. Evidence reports that microglia cells constitutively express CB1r at low levels, while CB2r are expressed at higher levels and are mainly modulated in relation to the cell activation state (Cabral, 2005; Carlisle et al., 2002; Ribeiro, Wen, Li, & Zhang, 2013). Consistently with previous findings, CB1r expression in BV‐2 cells resulted much lower than CB2r in basal condition (mean Ct ± SD, CB1r: 33.94 ± 1.47, CB2r: 21.33 ± 0.49 in 100 ng of BV‐2 CTRL cDNA; Figure S1), and not significantly modulated following a short LPS stimulus (−0.18‐fold LPS [2 hr] vs. CTRL group; data not shown). Based on these premises, we examined the CB2r expression in BV‐2 cells at different times of exposure to LPS. After 2 hr, CB2r transcript levels were reduced by about 9‐fold compared to the untreated control (Figure 2d). The downregulation lasted also at 6 and 24 hr (8.0 and 7.6‐fold lower, respectively) (*F*(3;16) = 132.368; *p* < .0001; Figure 2d), however, a significant difference was observed in CB2r mRNA expression between 2 and 24 hr (*p* = .031 vs. 2 hr LPS). The effect of LPS on CB2r protein levels, analyzed by means of western blotting, was consistent with what was observed for gene expression (Figure 2e). After 2 hr of LPS exposure, CB2r protein was 6.4‐fold reduced compared to the untreated control and persisted lower after 6 hr with respect to unstimulated cells (*F*(3;10) = 21.866; *p* < .0001; Figure 2e), while CB2r levels were not significantly different from the control group at 24 hr of LPS stimulation. Overall, our data depicted an early (2 hr) CB2r modulation in LPS‐induced activated microglial cells accompanied by a significant pro‐inflammatory cytokines release. Therefore, we choose to employ a short‐term stimulation with LPS in the following experiments.

### 3.3 CSE counteracts LPS‐induced microglial activation evaluated by the reduction of TNF‐α, IL‐6, and IL‐1β production

**FIGURE 3:**
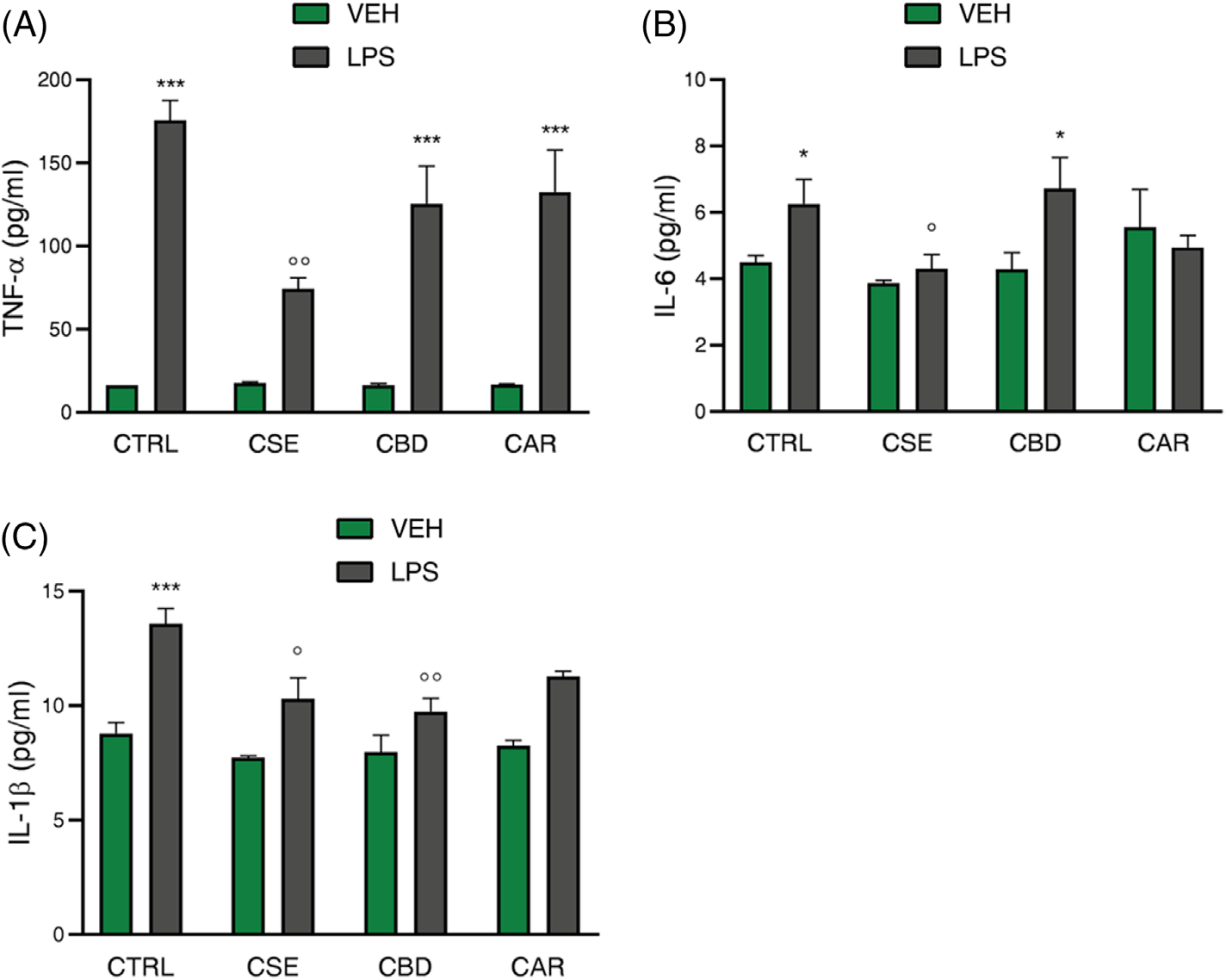
Effect of CSE, CBD, and CAR (1 μg/ml) treatment on TNF‐α (A), IL‐6 (B), and IL‐1β (C) release analyzed by ELISA assay in unstimulated and LPS‐stimulated BV‐2 cells (250 ng/ml). Each column represents mean ± SEM. Data were analyzed by one‐way analysis of variance followed by Tukey: *p < .05,***p < .001 versus CTRL group; °*p* < .05, °°*p* < .01 versus LPS (*n* = 6). CAR, β‐caryophyllene; CBD, cannabidiol; CSE, *Cannabis sativa* extract; LPS, lipopolysaccharide; SEM, standard error of the mean

Pro‐inflammatory cytokines represent one of the end‐effectors of microglia activation. To understand the pharmacological role of CSE on LPS‐induced microglia activation, cells were pre‐treated for 4 hr either with CSE or with the single constituents CBD and CAR, which are an inverse agonist and agonist of CBrs, respectively. According to viability assay, CSE, CBD, and CAR were used at 1 μg/ml concentration, which resulted in the maximum dose tested non‐cytotoxic for all the analyzed compounds (Figure S2a–c). Moreover, to exclude potential cytotoxicity resulting from the association of CSE, CBD, and CAR with LPS, cell viability was evaluated by 3‐(4,5‐dimethylthiazol‐2‐yl)‐2,5‐diphenyl tetrazolium bromide (MTT) assay in the presence of the endotoxin. The results showed that all compounds did not affect the viability of LPS‐stimulated microglial cells after 24 hr of treatment (Figure S2d). After 2 hr of LPS stimulation, an ELISA assay was used to measure the levels of the cytokines TNF‐α, IL‐6, and IL‐1β in BV‐2 cells. First, a pre‐treatment with CSE, CBD, and CAR failed to affect protein levels of the three cytokines considered in cells not exposed to LPS, and, as expected, exposure to LPS for 2 hr significantly increased TNF‐α, IL‐6, and IL‐1β levels above those of unstimulated controls (*F*(7;16) = 25.29 for TNF‐α; *F*(7;16) = 2.53 for IL‐6; *F*(7;16) = 12.75 for IL‐1β) (Figure 3a–c). The pre‐treatment with CSE significantly reduced the effect of LPS on the levels of TNF‐α, IL‐6, and IL‐1β. One‐way ANOVA revealed a main effect for TNF‐α (*p* < .01 vs. CTRL) and post‐hoc analysis showed that the effect of LPS on TNF‐α production was significantly reduced only in the presence of CSE with respect to cells exposed to LPS alone (0.42‐fold; *p* < .01 vs. LPS; Figure 3a). A 4‐hr pre‐treatment with CSE prevented LPS‐mediated induction of both IL‐6 (0.68‐fold; *p* < .05 vs. LPS; Figure 3b) and IL‐1β (0.76‐fold; *p* < .05 vs. LPS; Figure 3c). CBD was able to reduce IL‐1β protein levels in LPS stimulated cells as well (Figure 3c). CAR pre‐treatment did not show any significant effect on none of the tested cytokines. The inhibitory effect on LPS‐induced pro‐inflammatory cytokines production by microglia cells supported the potential anti‐inflammatory activity of CSE.

### 3.4 CSE, CBD, and CAR modulate LPS‐induced effects on CB2r protein but not mRNA expression

Several functional aspects were taken into account in this work to understand the CSE mechanism in LPS‐induced microglia response, such as the modulation of CB2r and nuclear peroxisome proliferator‐activated receptors γ (PPARγ). Anti‐inflammatory effects of endocannabinoids and phytocannabinoids in the brain might be mediated, at least in part, through activation of PPARγ (Iannotti & Vitale, 2021; Lago‐Fernandez, Zarzo‐Arias, Jagerovic, & Morales, 2021; O'Sullivan, 2007, 2016).

**FIGURE 4:**
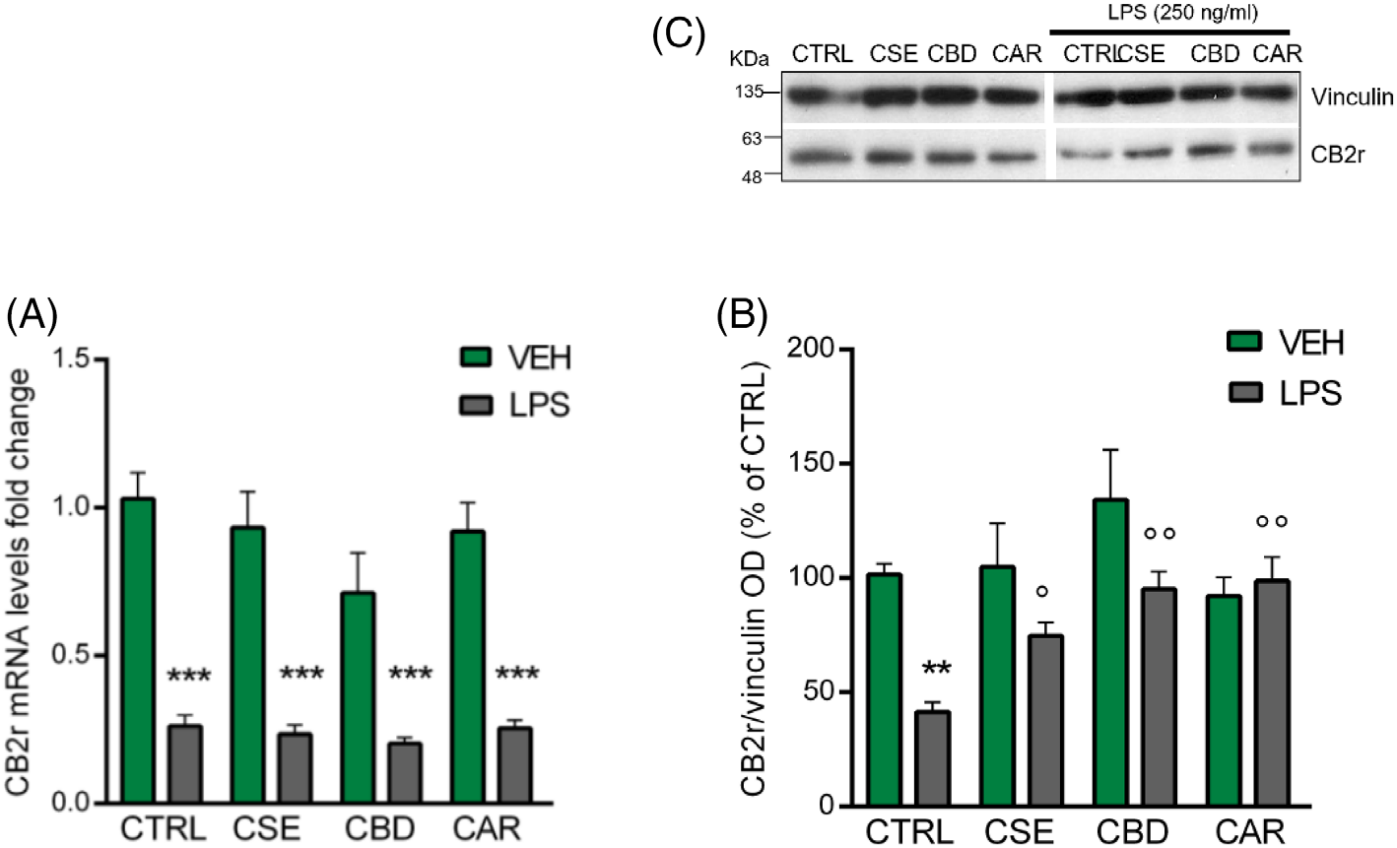
RT‐qPCR analysis of CB2r transcript (A) and protein (B) levels measured by western blot following the CSE, CBD, and CAR (1 μg/ml) treatments in unstimulated and LPS‐stimulated BV‐2 cells (250 ng/ml). (C) Immunoblot represents protein levels of CB2r and the endogenous target vinculin. Each column represents mean ± SEM. Data were analyzed by one‐way analysis of variance followed by Tukey: **p < .01, ***p < .0001, *versus* CTRL group; °*p* < .05, °°*p* < .01, *versus* LPS (*n* = 6 for mRNA, *n* = 4 for protein). CAR, β‐caryophyllene; CBD, cannabidiol; CSE, *Cannabis sativa* extract; LPS, lipopolysaccharide; SEM, standard error of the mean

By means of RT‐qPCR, we analyzed the CB2r and PPARγ mRNA levels in BV‐2 cells pre‐treated with CSE, CBD, and CAR for 4 hr and then stimulated 2 hr with LPS. The RT‐qPCR analysis of PPARγ mRNA levels highlighted that, in our model, CSE phytocomplex, but not CBD or CAR, significantly upregulated PPARγ transcript levels in unstimulated cells, while a 2 hr‐exposure to LPS did not affect its expression (Figure S3). In unstimulated cells, each of the tested compounds did not affect the expression levels of CB2r mRNA (Figure 4a). Significant downregulation of the CB2r mRNA levels was observed in all the groups exposed to LPS (*F*(7;45) = 48.52; *p* < .001; Figure 4a). Indeed, CSE, CBD, or CAR were not able to modify the effect of the immune stimulation on CB2r expression. As for the mRNA levels, one‐way ANOVA revealed a main effect of the inflammatory stimulus for CB2r protein expression: a 2 hr‐exposure to LPS significantly reduced CB2r protein levels compared to control cells (*F*(7;20) = 9.02; *p* < .01; Figure 4b). Post‐hoc analysis revealed that none of the pre‐treatments had a significant effect on CB2r protein levels in unstimulated microglia. By comparing LPS‐stimulated cells, both CBr selective ligands CBD and CAR were able to significantly counteract the LPS‐induced downregulation of CB2r protein (*p* = .001 vs. LPS; Figure 4c). An effect on preventing LPS‐induced CB2r decrease was observed also for CSE (*p* = .022 vs. LPS; Figure 4c). Interestingly, these results suggest that the modulation of CB2r could play a role in CSE inhibition of LPS‐induced pro‐inflammatory cytokines production. At the same time, the different effect between *C*. *sativa* phytocomplex and the single compounds (CBD and CAR) in counteracting LPS‐induced microglial response implies that other intracellular mechanisms could underlie CSE activity.

### 3.5 CSE modulates the expression of AEA and 2‐AG metabolic enzymes in LPS‐stimulated BV‐2 cells

Microglia respond to activation by producing AEA and 2‐AG (Stella, 2009), two major endogenous agonists of CB1r and CB2r, which demonstrated immunomodulatory activity and neuroprotective properties (Chang, Lee, & Lin, 2001; Turcotte, Chouinard, Lefebvre, & Flamand, 2015).

Considering that, we aimed of investigating the potential involvement of AEA and 2‐AG in LPS‐induced microglia response and the possible activity of CSE, CBD, and CAR in modulating the endocannabinoids system. To do this, we analyzed the expression of the enzymes responsible for the 2‐AG and AEA metabolism. The endogenous levels of 2‐AG are controlled by the balance of biosynthetic and degrading enzymes activity: diacylglycerol lipase (DAGL) metabolizes diacylglycerol to produce 2‐AG, which in turn is inactivated by monoacylglycerol lipase (MAGL) hydrolysis into arachidonic acid (Alhouayek et al., 2014; Tanaka, Sackett, & Zhang, 2020).

Concerning AEA production, there are multiple pathways responsible for its biosynthesis; *N*‐acyl phosphatidylethanolamine phospholipase d (NAPE‐PLD) is considered the main biosynthetic enzyme catalysing the cleavage of *N*‐acylethanolamine from *N*‐arachidonoyl‐phosphatidylethanolamine, while the principal degrading enzyme of AEA is the fatty acid amide hydrolase (FAAH) which also contributes to hydrolyse 2‐AG (Di Marzo, Melck, Bisogno, & De Petrocellis, 1998).

By means of RT‐qPCR we measured the mRNA levels of DAGL‐β, the microglia‐specific synthesizing enzyme for 2‐AG, NAPE‐PLD, and FAAH in BV‐2 cells pre‐treated for 4 hr with CSE, CBD, or CAR and then stimulated with LPS for 2 hr.

Consistently with previous findings, the expression of MAGL mRNA was very low in BV‐2 cells (Muccioli et al., 2007), as confirmed by our analysis (mean Ct ± SD, MAGL: 33.21 ± 0.37 in 50 ng of BV‐2 CTRL cDNA).

**FIGURE 5:**
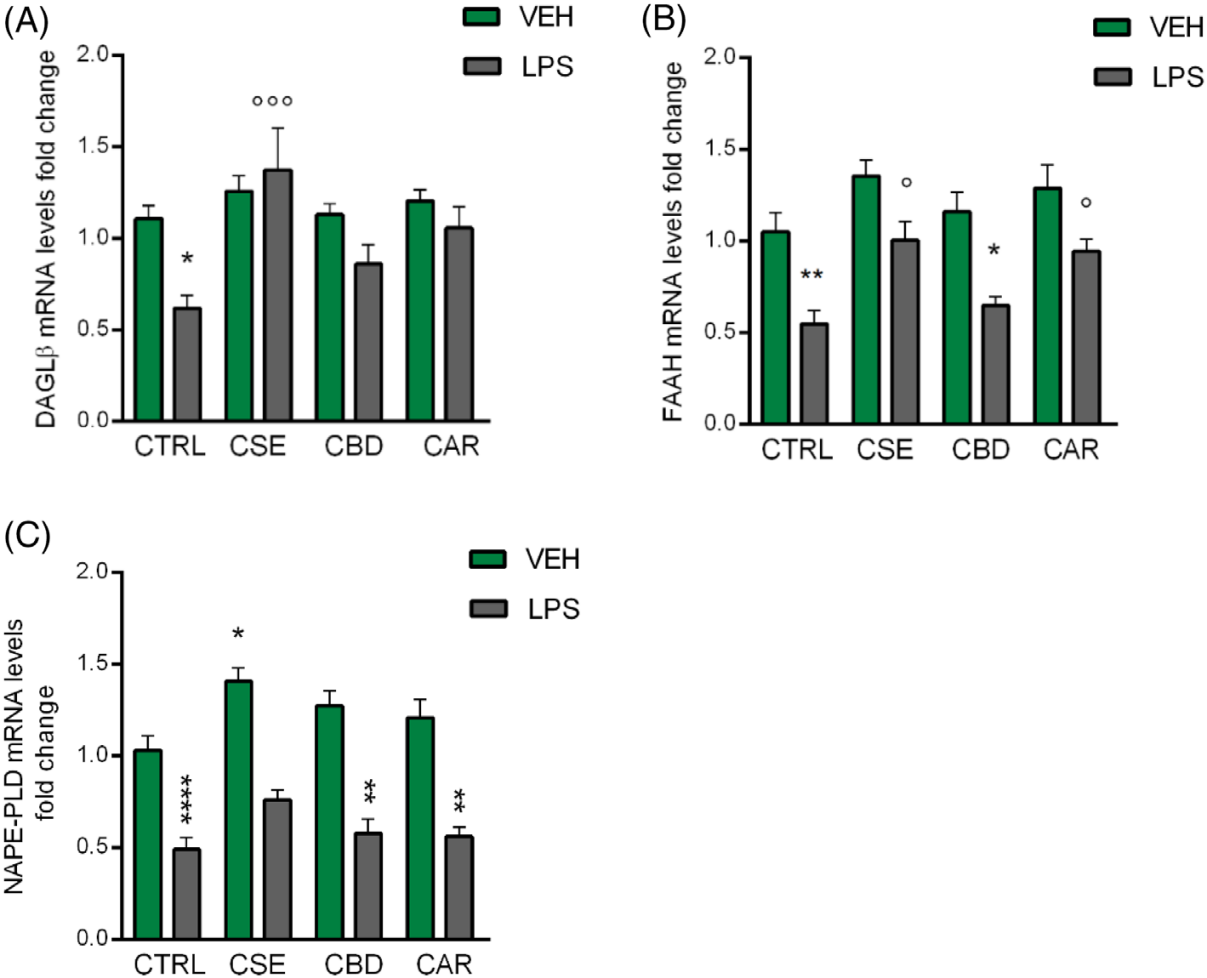
RT‐qPCR analysis of DAGL‐β (A) FAAH (B) and NAPE‐PLD (C) transcripts in BV‐2 cells following the CSE, CBD, and CAR (1 μg/ml) treatments in unstimulated and LPS‐stimulated BV‐2 cells (250 ng/ml). Each column represents mean ± SEM. Data were analyzed by one‐way analysis of variance followed by Tukey: *p < .05, **p < .01, ****p < .0001 *versus* CTRL group; °*p* < .05, °°°°*p* < .0001 *versus* LPS (*n* = 10). CAR, β‐caryophyllene; CBD, cannabidiol; CSE, *Cannabis sativa* extract; DAGL, diacylglycerol lipase; FAAH, fatty acid amide hydrolase; LPS, lipopolysaccharide; NAPE‐PLD, *N*‐acyl phosphatidylethanolamine phospholipase d **;** SEM**,** standard error of the mean

Treatment with CSE, CBD, and CAR did not alter DAGL‐β and FAAH transcript levels (Figure 5a,b), while NAPE‐PLD mRNA expression was increased only by CSE in unstimulated cells (*p* = .011 *vs*. CTRL; Figure 5c). Upon stimulation with LPS, a significant downregulation of DAGL‐β mRNA levels was observed in BV‐2 cells compared to controls (*F*(7;65) = 4.72; *p* < .05 vs. CTRL; Figure 5a). Post‐hoc analysis showed that CSE and CAR pre‐treatments were able to counteract the LPS‐induced downregulation of DAGL‐β, but this effect was statistically significant only for the phytocomplex (*p* = .0004 *vs*. LPS, Figure 5a).

Following LPS exposure, one‐way ANOVA highlighted a significant decrease of both FAAH and NAPE‐PLD mRNA levels compared to controls (*F*(7;67) = 10.41, *p* < .01 for FAAH; *F*(7;66) = 23.83; *p* < .0001 for NAPE‐PLD; Figure 5b,c) and post‐hoc analysis revealed the effect of CSE (*p* = .042 *vs*. LPS) and CAR (*p* = .026 *vs*. LPS) in preventing LPS‐induced downregulation of FAAH (Figure 5b). Conversely, CSE, CBD, and CAR were not able to revert the effect of the immune stimulation on NAPE‐PLD mRNA expression (Figure 5c).

These results showed that an acute LPS‐induced inflammatory condition inhibited microglia metabolic enzymes of AEA and 2‐AG at the transcriptional level, while CSE phytocomplex could preserve their expression. This hinted a complementary mechanism, besides the CBrs modulation, underlying the phytocomplex activity on the ECS response to microglial inflammatory stress.

### 3.6 CSE inhibits JNK and p38, but not ERK activation in LPS‐stimulated BV‐2 cells

**FIGURE 6:**
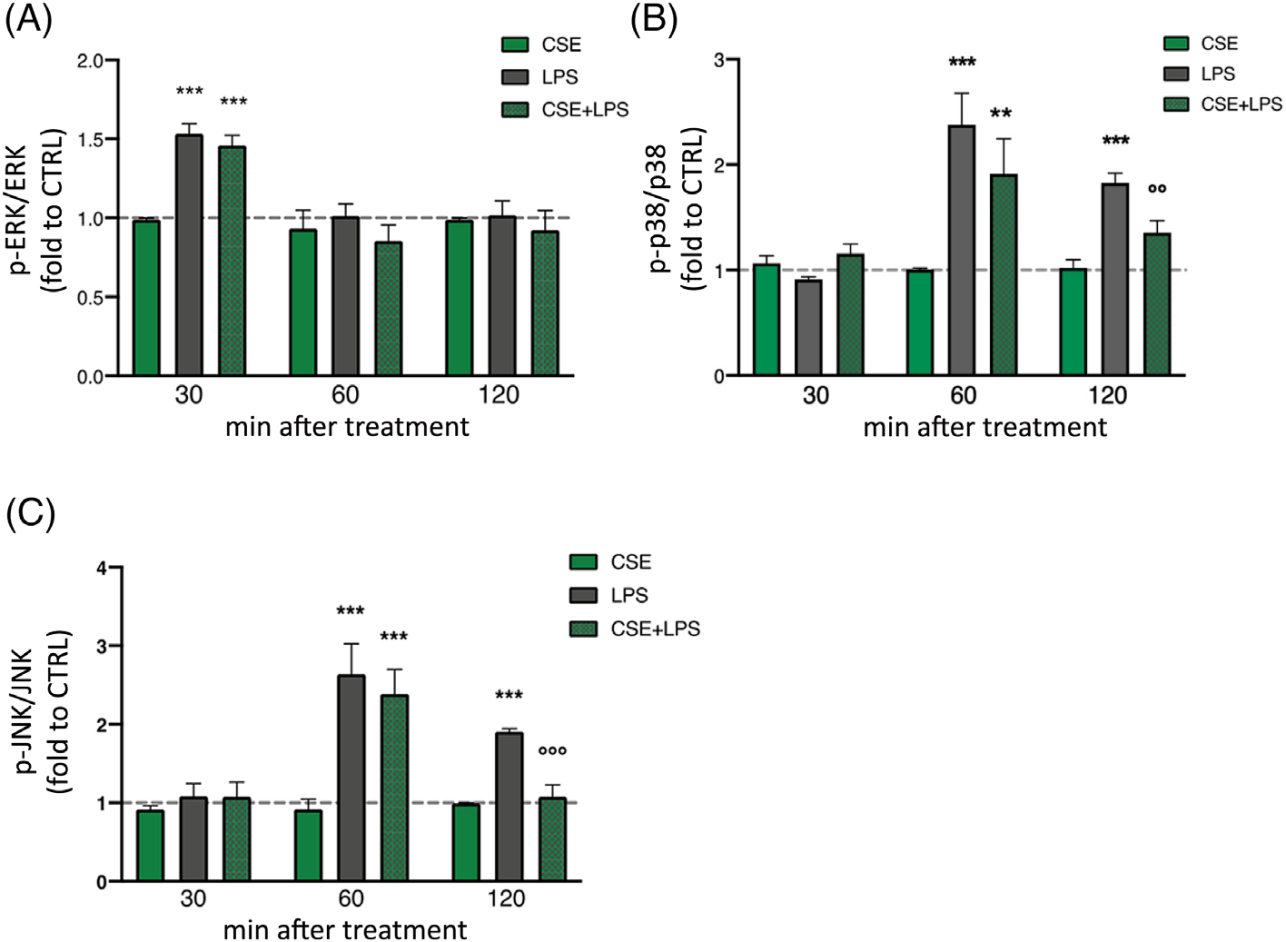
Effect of CSE (1 μg/ml) in basal condition and after 30, 60, and 120 min of LPS exposure (250 ng/ml) on ERK (A) p38 (B) and JNK (C) phosphorylation in BV‐2 cells. Levels of total and phosphorylated protein were analyzed by ELISA assay. Each column represents mean ± SEM. The dashed line represents the CTRL value set at 1. Data were analyzed by one‐way analysis of variance followed by Tukey: ***p < .001 *versus* CTRL group; °°*p* < .01, °°°*p* < .001 *versus* LPS (*n* = 3). CSE, *Cannabis sativa* extract; ERK, extracellular signal‐regulated kinases; JNK, c‐Jun N‐terminal kinases; LPS, lipopolysaccharide; SEM, standard error of the mean

MAPKs have been investigated as intracellular mechanisms involved in the regulation of the cellular inflammatory response. A large number of studies pointed out ERK, p38, and JNK MAPKs activity as responsible for most cellular responses to external stress signals and crucial for the regulation of transcription and translation of inflammation mediators (Alboni et al., 2014; Edelmayer, Brederson, Jarvis, & Bitner, 2014; Falcicchia, Tozzi, Arancio, Watterson, & Origlia, 2020; Kaminska, 2005; Kaminska, Gozdz, Zawadzka, Ellert‐Miklaszewska, & Lipko, 2009; Kim & Choi, 2010). Given the peculiar ability of CSE in modulating cytokines release, endocannabinoids metabolism, and CB2r expression induced by a 2‐hr LPS exposure, we investigated whether this effect could be associated with an early regulation of MAPKs activity by measuring the phosphorylation of ERK, p38, and JNK MAPKs through ELISA dosage in a short time course of LPS stimulation (30, 60, and 120 min). After LPS stimulation, ERK activation peaked at 30 min with an increase of 1.46‐fold compared to the control cells, while no difference between stimulated and unstimulated microglia was observed at the later time points (*F*(3;8) = 113.00; *p* < .001; Figure 6a). LPS exposure affected p38 and JNK activation in a similar way: 60 min of stimulation caused a significant increase in phosphorylation levels of both kinases with respect to control cells; this effect dampened but was still present at 120 min (*F*(3;8) = 56.95; *p* < .001 for p38; *F*(3;8) = 97.90; *p* < .001 for JNK; Figure 6b,c). Post‐hoc analysis revealed that CSE pre‐treatment alone did not alter the phosphorylation levels of any tested MAPKs at all times examined. When stimulated with LPS, CSE pre‐treatment failed to affect ERK activation. On the other hand, CSE significantly reduced p38 (*p* < .01 vs. LPS, Figure 6b) and JNK phosphorylation after 120 min (*p* < .001vs. LPS; Figure 6c).

Based on these results, we may suppose that the CSE effect on microglia inflammatory response could be even mediated at intracellular level by the phosphorylation of p38 and JNK MAPKs.

### 3.7 CSE inhibits NF‐κB nuclear translocation in LPS‐stimulated BV‐2 cells

**FIGURE 7:**
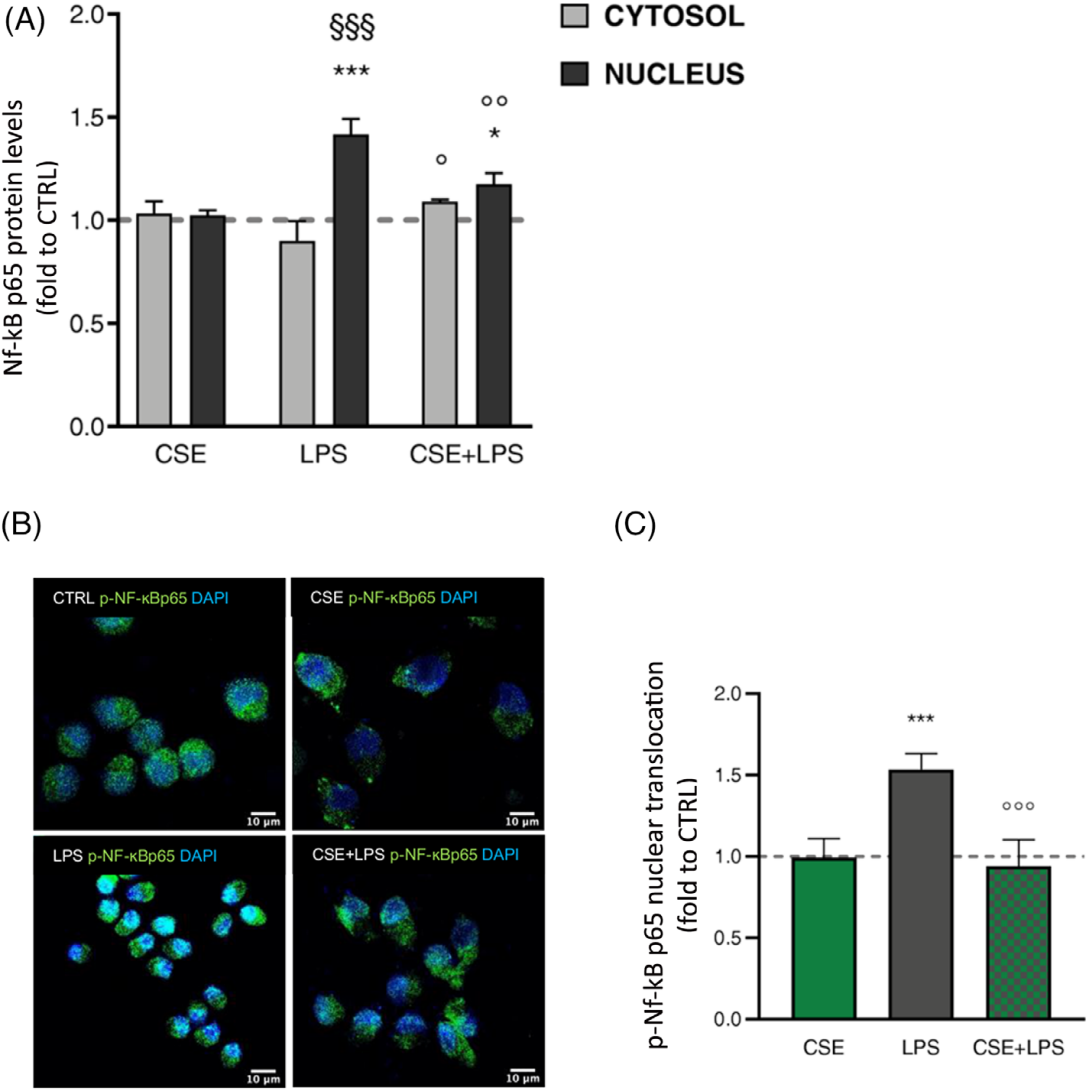
(A) Cytosol and nucleus dosage of total NF‐κb p65 protein levels by ELISA assay following treatment with CSE and LPS stimulation for 60 min. Each column represents mean ± SEM. Data were analyzed by one‐way analysis of variance followed by Tukey: ***p < .001 *versus* CTRL group; °*p* < .05, °°*p* < .01, °°°*p* < .001 *versus* LPS; ^§§§^ *p* < .001 *versus* LPS cytosol (*n* = 6). (B) Immunostaining of NF‐κB phospho‐p65 (Ser536) nuclear translocation (green) in BV‐2 cells untreated (CTRL) or CSE‐treated (1 μg/ml), and after LPS (250 ng/ml) exposure for 60 min (CSE + LPS). (C) Histogram depicts immunofluorescence signal quantification by ImageJ. The dashed line represents the CTRL value set at 1. One‐way analysis of variance followed by Tukey: ***p < .001 *versus* CTRL group, °°°*p* < .001 *versus* LPS (*n* = 3). CSE, *Cannabis sativa* extract; LPS, lipopolysaccharide; SEM, standard error of the mean

The transcription factor NF‐κB regulates the expression of a large array of genes involved in immune and inflammatory responses, including cytokines. NF‐κB protein is ubiquitously expressed in the cytoplasm where it is retained latent by IκB inhibitory proteins through the interaction with NF‐κB p65 subunit. In response to a variety of stimuli, IκB inhibitory proteins are degraded and NF‐κB translocates in the nucleus where NF‐κB‐dimer, p50 and p65, can bind DNA site promoters (Buss et al., 2004; Li, Zhao, Lin, Gong, & An, 2019; Shih, Wang, & Yang, 2015). Therefore, to examine the role of NF‐κB in CSE activity, we measured total NF‐κB p65 protein levels by ELISA dosage in both cytosol and nuclear fractions of BV‐2 cells treated 4 hr with CSE before LPS exposure for 60 min. After LPS stimulation, NF‐κB p65 protein levels significantly increased in microglia nucleus compared to control cells (*F*(2;6) = 23.14; *p* < .001 *vs*. CTRL; Figure 7a). CSE pre‐treatment alone did not alter NF‐κB p65 protein levels in both cytosol and nuclear fractions, whereas following immune stimulus, CSE significantly reduced NF‐κB p65 protein levels in microglia nuclei (*p* < .01 *vs*. LPS). Although the nuclear translocation of NF‐κB is essential for its activation, post‐translational modifications of NF‐κB subunits, such as phosphorylation, contribute significantly to the activity of NF‐κB. Evidence showed that LPS is able to induce the phosphorylation on Ser536 residue of NF‐κB p65 regulating its transactivation in monocytes and macrophages (Bagaev et al., 2019; Viatour, Merville, Bours, & Chariot, 2005; Yang, Tang, Guan, & Wang, 2003). Considering that, we evaluated nuclear NF‐κB p‐p65 levels by immunostaining in BV‐2 cells treated 4 hr with CSE and then exposed to LPS for 60 min. Immunofluorescence images did not reveal NF‐κB p‐p65 positive nuclei in control and CSE‐treated cells. Consistently with our previous study (Borgonetti et al., 2020), we detected marked staining for NF‐κB p‐p65 in BV‐2 nuclei after 60 min of LPS stimulation (Figure 7b). In LPS‐stimulated cells, CSE pre‐treatment was able to completely inhibit the nuclear translocation of NF‐κB p‐p65 displayed by the loss of NF‐κB p‐p65 staining at nuclear level (Figure 7b). Quantification of staining intensity confirmed a significant increase in phosphorylation levels of NF‐κB p65 subunit in the nucleus of LPS‐stimulated microglia compared to unstimulated cells (*F*(3;8) = 29.67; *p* < .001; Figure 7c). Post‐hoc analysis corroborated the effect of CSE in reducing NF‐κB p‐p65 levels induced by LPS in the BV‐2 nucleus (Figure 7c). In support of previous findings, CSE activity on microglia inflammatory response involves the NF‐κB signaling pathway.

### 3.8 CSE reduces intracellular oxygen species (ROS) production in LPS‐stimulated BV‐2 cells

**FIGURE 8:**
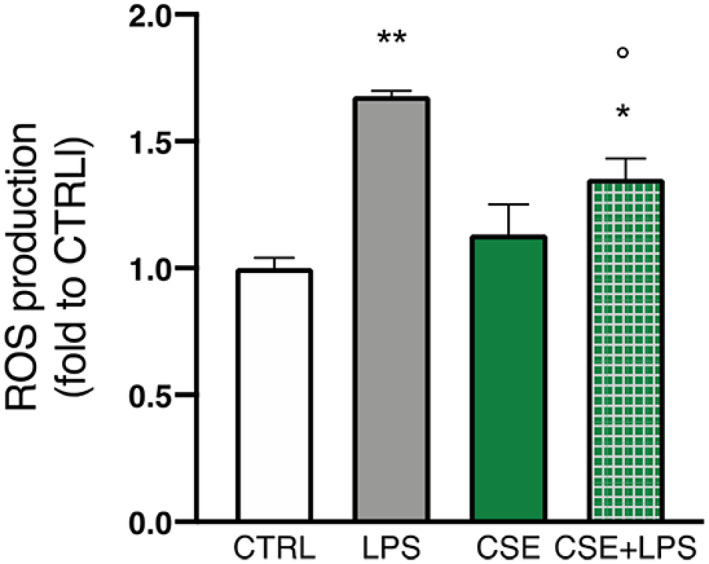
Effect of CSE (1 μg/ml) in basal condition and after 30, 60, and 120 min of LPS exposure (250 ng/ml) on ROS release in BV‐2 cells measured by fluorometric intracellular assay. Each column represents mean ± SEM. Data were analyzed by one‐way analysis of variance followed by Tukey: *p < .05, **p < .01 *versus* CTRL group, °*p* < .05 *versus* LPS (*n* = 6). CSE, *Cannabis sativa* extract; LPS, lipopolysaccharide; SEM, standard error of the mean

The antioxidant and antiradical activity of *C*. *sativa* constituents have been already considered regarding their neuroprotective activity (Borgonetti et al., 2019). Based on this premise, the effect of CSE on ROS production was investigated by means of the fluorometric intracellular assay in BV‐2 cells pre‐treated 4 hr with CSE and then stimulated 2 hr with LPS. The statistical analysis highlighted that LPS exposure induced a significant increase of ROS in microglia compared to untreated cells (*F*(3;8) = 15.81; *p* < .01; Figure 8). In basal conditions, CSE treatment did not affect ROS production with respect to control cells. Post‐hoc analysis indicated that, in the presence of LPS stimulation, pre‐treatment with CSE counteracted the ROS production (1.2‐fold; *p* < .05 *vs*. LPS; Figure 8). These results allowed us to confirm the activity of CSE in the modulation of oxidative stress response and the related ROS production. To evaluate the direct radical scavenging activity of CSE, the cell‐free DPPH test was then carried out. CSE showed a moderate direct scavenger activity by a measured IC_50_ of 190.20 μg/ml. Effective concentration in DPPH scavenging activity resulted very higher compared to the ROS inhibiting effect in LPS‐stimulated BV‐2 cells, thus suggesting that CSE mostly works by modulating intracellular antioxidant defense.

## 4 DISCUSSION

The purpose of the current study was to explore the molecular mechanisms underlying the protective activity of a non‐psychotropic *C*. *sativa* extract in an *in vitro* model of neuroinflammation achieved by the acute administration of the endotoxin LPS in BV‐2 microglia cells. Growing evidence demonstrates the involvement of the ECS in mediating the inflammatory cascade and microglial activation. In this context, this work is focused on *C*. *sativa*, since this medicinal plant is the main source of exogenous CBr ligands and because phytocannabinoids, terpenes, and flavonoids were all proven to affect microglia intracellular processes regulating inflammatory response through multitarget mechanisms (Stasiłowicz, Tomala, Podolak, & Cielecka‐Piontek, 2021). The first key point of the current study was the production and the chemical characterization of a non‐psychotropic *C*. *sativa* variety, rich in CBD and terpenes. By means of an automated extraction procedure, we obtained an extract (CSE) from non‐psychotropic *C*. *sativa* inflorescences (var. Carmagnola) rich in CBD (21%) (CBD:THC ratio equal to 42:1), CBG (1.0%) and with a high content of total terpenes (>12.5%), mainly represented by CAR and caryophyllene oxide. Our approach is relevant because, similarly to many other herbal products not enlisted in official Pharmacopoeias, one of the most important limitations for the pharmacological use of non‐psychotropic *C*. *sativa* varieties is represented by the high variability of phytochemicals in different herbal preparations, namely CBD level, CBD:THC ratio, and terpenes content. The importance of employing a standardized extract has been corroborated by a recent large‐scale study where a comparison of a wide variety of hemp in an in vitro 3D model of oral, airway, and intestinal tissues (Wang et al., 2020), demonstrated a positive correlation between biological activity, CBD, and terpenes content. Secondly, we found that (2 hr) LPS‐stimulation provided a strong downregulation of CB2r, besides an impaired expression of the endogenous cannabinoids metabolic enzymes, and a massive increase of pro‐inflammatory cytokines in BV‐2 microglial cells.

CSE was effective in reducing LPS‐induced TNF‐α, IL‐6, and IL‐1β production. To the best of our knowledge, this is the first study that reports the anti‐inflammatory effect of a standardized non‐psychotropic *C*. *sativa* extract in LPS‐stimulated microglia cells. Our previous work similarly highlighted the inhibitory effect of a *C*. *sativa* extract, containing 21.6% CBD and 7.1% terpenes, on IL‐6 release in LPS‐stimulated PBMC (Rigillo et al., 2019). The beneficial effects of full‐spectrum cannabis extract were confirmed also in different animal models of chronic inflammatory conditions, such as neuropathic pain (Comelli, Giagnoni, Bettoni, Colleoni, & Costa, 2008).

The two main individual constituents of CSE, namely CBD and CAR, were also tested at the same concentration of CSE but they showed lower activity in inhibiting LPS‐induced cytokines production in BV‐2 cells. CBD (1 μg/ml) inhibited IL‐1β release likewise to CSE, but it showed an almost null effect on TNF‐α and IL‐6 production. Data are only partially consistent with those reported by dos‐Santos‐Pereira, Guimarães, Del‐Bel, Raisman‐Vozari, and Michel (2020), who described the effect of 1–10 μM CBD (0.314–3.14 μg/ml) in hindering LPS‐induced microglial inflammation by suppressing IL‐1β and TNF‐α production. Of note, in stimulated BV‐2 cells CAR was found to be ineffective toward cytokines upregulation. Previous data indicated a good activity of CAR, tested at similar concentrations, in inhibiting pro‐inflammatory cytokines release in LPS‐stimulated mice primary microglia cells (0.2–25 μM) (Askari & Shafiee‐Nick, 2019), and in hypoxia‐induced stress in BV‐2 cells (5 μM) (Guo, Mou, Huang, Xiong, & Li, 2014) but, in both studies, CAR treatment was prolonged up to 24 hr.

We found that the CBD‐enriched non‐psychotropic *C*. *sativa* extract was able to modulate the transcription of endocannabinoids‐metabolism enzymes in BV‐2 microglia cells both in basal and inflammatory conditions. Indeed, the analysis of the transcriptional levels of synthesizing and degrading enzymes responsible for 2‐AG and AEA metabolism highlighted the effectiveness of CSE in positively upregulating the AEA‐synthesizing enzyme, NAPE‐PLD, in physiological conditions. This aspect is noteworthy considering that AEA plays an antiinflammatory role in activated microglia (Correa et al., 2010; Malek, Popiolek‐Barczyk, Mika, Przewlocka, & Starowicz, 2015; Pflüger‐Müller et al., 2020; Sedeighzadeh, Galehdari, Tabandeh, Shamsara, & Roohbakhsh, 2021). The most relevant finding was the effect of CSE in counteracting the LPS‐induced downregulation of metabolic enzymes, possibly contributing to CSE in maintaining high levels of the endocannabinoids 2‐AG and AEA, whose neuroprotective role is known (Eljaschewitsch et al., 2006; Panikashvili et al., 2001; Papageorgis et al., 2011). Convincing findings have shown that activation of endocannabinoid signaling can repress microglial activation and ameliorate neurodegeneration in several neurological diseases (Tanaka et al., 2020).

Interestingly, CBD was devoid of effect on the expression of the enzyme responsible for endocannabinoids metabolism in LPS‐stimulated BV‐2, whereas CAR was only significantly effective in counteracting FAAH downregulation. On the other hand, CAR and CBD significantly reverted the LPS‐induced CB2r protein downregulation at 2 hr, showing a protective mechanism through the direct modulation of the ECS.

Indeed, sturdy evidence suggests that targeting the ECS with CB2r ligands may lead to a mitigation of microglial inflammatory insult, both *in vivo*, in LPS‐induced neuroinflammation in mice (Sahu et al., 2019), and in cellular models, as reported in human periodontal ligament fibroblasts stimulated with LPS (Abidi et al., 2018), or in LPS‐stimulated macrophages, and in mesenchymal stromal cells (Ruhl et al., 2020). Also, CSE reverted the LPS‐induced CB2r protein decrease, but to a lesser extent when compared to CBD and CAR, suggesting that the strong activity of *C*. *sativa* phytocomplex in preventing cytokines release is only partially modulated by the ECS. The modulation of CB2r protein expression did not correlate with mRNA transcription levels. This is most likely to be attributed to a greater effect of LPS on CB2r protein levels rather than its gene transcription, consistent with the dominant role of protein expression regulation following stress stimuli (Cheng et al., 2016). Our data could not fully explain if the co‐presence of a CB2r full agonist (CAR) and an inverse agonist (CBD) in CSE produced a negative interaction on LPS‐induced modulation of CBr or, more plausibly, CBD and CAR content in CSE (21 and 3% ca. respectively, equal to 210 and 30 ng/ml in CSE) was too low to display a greater effect against the decrease of CB2r following immune stimulation in BV‐2 cells (Anil et al., 2021; dos‐Santos‐Pereira et al., 2020; Guo et al., 2014).

We examined PPARγ signaling as being known to mediate the anti‐inflammatory activity of CBD, as well as those of endocannabinoids 2‐AG and AEA (O'Sullivan, 2007, 2016). Despite this, in our *in vitro* model, PPARγ expression was not affected by LPS stimulus at the transcriptional level and it cannot be considered an involved mechanism. Furthermore, CSE phytocomplex, but not CBD or CAR, positively upregulated PPARγ expression. Distinct signaling pathways, beside ECS and PPARγ modulation, have been considered to explain the cannabinoid‐mediated regulation of LPS‐induced activation of microglia: the involvement of MAPKs and JAK/STAT pathways was confirmed for CBD and THC, as well as NF‐κB and AP‐1 negative regulation. Also the modulation of the oxidative stress response Nrf2/ATF4‐Trib3 pathway was considered to underlie the anti‐inflammatory effect of CBD (Juknat et al., 2013). We observed that in BV‐2 cells a short LPS stimulation caused the NF‐κB nuclear translocation (peak at 60 min) and MAPKs phosphorylation. CSE targeted MAPKs pathway by reducing JNK and p38 activation in stimulated microglial cells; moreover, an evident inhibition of NF‐κB nuclear translocation was produced by CSE treatment in LPS‐stimulated BV‐2 cells. On the other hand, CSE did not modulate the p‐ERK level. Results were intriguing and depicted a protective effect of cannabinoids already observed in an *in vivo* model of acute lung inflammatory injury induced by Paraquat. The CB2r agonist JWH133 was able to ameliorate Paraquat‐induced lung histopathological inflammatory changes by reducing MAPKs activation and by attenuating NF‐κB signal transduction (Liu et al., 2014). These results clearly showed that the effect of CSE on cytokine production in LPS‐stimulated BV‐2 is mostly related to the inhibition of NF‐κB nuclear translocation and this is linked in turn to the modulation of JNK/p38 MAPKs. Thus, the protective effect of CSE on inflammatory cascade could not be connected only to the peculiar activation of CB2r. It is most likely that CSE phytocomplex may also target an upstream less specific signaling pathway capable of attenuating LPS‐induced inflammatory response. This upstream mechanism triggered by CSE could correlate, at least in part, with the antioxidant and ROS quenching activity exerted by CSE. Microglial cells mediate oxidative stress in response to pathogen‐ or damage‐associated molecules by producing ROS and other reactive compounds. ROS act as both a signaling molecule and as a mediator of inflammation (Mittal, Siddiqui, Tran, Reddy, & Malik, 2014). Moreover, several studies report that microglial ROS synthesis sustains oxidative stress associated with neurodegeneration (Simpson & Oliver, 2020). It is known that LPS stimulation leads to intracellular ROS production in BV‐2 cells (Corsi, Momo Dongmo, & Avallone, 2015) and that ROS are a key activator of both MAPKs and NF‐κB transduction (Zhang et al., 2016); thus targeting ROS scavenging is a known mechanism to attenuate microglial inflammatory response. Indeed, we demonstrated that CSE was effective in reducing LPS‐generated high ROS levels. The weak activity exerted by CSE in the DPPH scavenging test suggested that the antioxidant property of CSE may depend on the modulation of intracellular antioxidant mechanisms.

## 5 CONCLUSIONS

Cannabinoids are currently considered to be promising compounds to treat neuroinflammation‐related diseases, as well as for the modulation of the ECS. In particular, CB2r activation represents a target for the mitigation of microglial inflammatory response. *C*. *sativa* L. is the most important natural source of exogenous cannabinoids that, among its non‐psychotropic varieties with low content of THC, are represented by the CB2r inverse agonist, CBD, and the full agonist, CAR, but also by other minor phytocannabinoids, together with terpenoids. Many of these molecules may target distinct inflammatory pathways proposing the positive role of the *C*. *sativa* phytocomplex. The current study describes for the first time that a chemically characterized *C*. *sativa* extract, obtained from EU authorized non‐psychotropic variety, displays a marked activity in attenuating pro‐inflammatory cytokines synthesis in activated microglial cells with higher efficacy than CBD and CAR at the same concentration. In this model, the protective effect of the CSE phytocomplex depends on intracellular mechanisms at a different level: the regulation of endogenous cannabinoids metabolism, ROS scavenging activity, the inhibition of JNK/p38 activation, NF‐κB signaling, and partially the modulation of CB2r. Despite further investigations being needed to better elucidate synergistic or antagonistic effects of the different constituents, these data suggested that the multitarget mechanism of *C*. *sativa* extracts could be considered as an interesting and novel therapeutic approach in the challenging field of neuroinflammation and related diseases.

## CONFLICT OF INTEREST

The authors declare no potential conflict of interest.

## AUTHOR CONTRIBUTIONS

**Marco Biagi**: Conceptualization; manuscript drafting. **Giovanna Rigillo**: Conceptualization; in vitro experiments; molecular analysis; statistical analysis; manuscript drafting; manuscript revision and editing. **Giovanni Isoldi**: Extract production; chemical analysis. **Federica Pellati**: Chemical analysis. **Vittoria Borgonetti**: In vitro experiments; molecular analysis; statistical analysis; manuscript drafting. **Paolo Governa**: In vitro experiments; molecular analysis; statistical analysis; manuscript drafting. **Cristina Benatti**: Molecular analysis; statistical analysis; manuscript revision and editing. **Silvia Alboni**: Manuscript revision and editing. **Fabrizio Manetti**: Manuscript revision and editing; resources and supervision. **Monica Montopoli**: Manuscript revision and editing. **Nicoletta Galeotti**: Resources and supervision. **Elisabetta Miraldi**: Resources and supervision. **Fabio Tascedda**: Resources and supervision. All authors have approved the final version submitted for publication.

## Supporting information


**Figure S1.** Analysis of the CB2r/CB1r mRNA ratio in BV‐2 microglia cells analyzed by means of RT‐qPCR. CB2r mRNA expression (Mean Ct±SD, CBr1: 33.94±1.47, CBr2: 21.33±0.49) in 100 ng of BV‐2 CTRL cDNA.
**Figure S2**. Cell viability of BV‐2 cells was tested by CCK‐8 assay after a 24 h treatment at the concentrations 0.01‐0.1‐1‐10 μg/mL of CSE (a), CBD (b), and CAR (c) respectively. Each column represents mean ± S.E.M. Data were analyzed by one‐way ANOVA: ***p<0.001, *p<0.05 *vs* CTRL group; (n = 6). (d) BV‐2 cell viability was tested by MTT assay following a 24‐hr cotreatment of LPS (250 ng/mL) with CSE, CBD, and CAR (1 μg/mL) respectively. Each column represents mean ± S.E.M. Data were analyzed by one‐way ANOVA: (n.s.) no significant differences were observed vs CTRL group; (n = 6).
**Figure S3**. RT‐qPCR analysis of PPARγ mRNA levels in BV‐2 cells following the CSE, CBD and CAR (1 μg/mL) treatments in unstimulated and LPS‐stimulated BV‐2 cells (250 ng/mL). Each column represents mean ± S.E.M. Data were analyzed by one‐way ANOVA followed by Tukey: *p<0.05 vs CTRL group (n = 5).

**Appendix S1**. Supporting information.

## Data Availability

The data that support the findings of this study are available from the corresponding author upon reasonable request.
