# Supplementary material for: Non‐psychotropic Cannabis sativa L. phytocomplex modulates microglial inflammatory response through CB2 receptors‐, endocannabinoids‐, and NF‐κB‐mediated signaling
Source: Phytother Res. 2022 Apr 8;36(5):2246–63. doi: 10.1002/ptr.7458 (PMC9325551; doi:10.1002/ptr.7458)

Supplementary information

1) Cell viability

Cell viability tests were performed by Cell Counting Kit-8 (CCK-8, Merck KGaA) or using 3-(4,5-dimethylthiazol-2-yl)-2,5-diphenyl tetrazolium bromide (MTT) assay, to exclude cytotoxic effects of the extract and its main constituent alone or in association with the endotoxin LPS. BV-2 cells ($5x10^4$ cells in 100 µL of medium) were cultured in 96-well plates. Single treatments were performed by incubating BV-2 cells with CSE, CBD or CAR (0.01-0.1-1-10 µg/mL) and the vehicle for 4 h. Then, CCK-8 solution was added to each well and incubated at 37 °C, 5% CO_2 for 60 min. Cell viability was calculated by measuring the absorbance at 450 nm. Co-treatments were performed by incubating BV-2 cells with CSE, CBD or CAR (1 µg/mL) in association with LPS (250 ng/mL) respectively for 24 h. MTT solution (5 mg/mL, Merck KGaA) was added to each well and incubated at 37 °C, 5% CO_2 for 4h, then formazan crystals were dissolved in 150 μL acid isopropanol (0.1 N HCl in isopropanol) and the absorbance was measured at 570 nm and at 620 nm as reference wavelengths. All experiments were performed in sextuplicate in three independent cultures. Results were normalized to control group.

2) Transcript and sequence of each primer used in Real-Time PCR.

Transcript	NCBI GenBank	Primer sequence
***Mus musculus* peroxisome proliferator activated receptor gamma (*PPARγ*)**	*NM_011146*	Fw CAGGCTTCCACTATGGAGTTC<br>Rv GGCAGTTAAGATCACACCTATCA

Supplementary Table 1: semi-quantitative data of the volatile compounds identified in *Cannabis sativa* L. extract by GC analysis. Data are expressed as % relative peak area ± SD (n = 3).

Peak number	Compound	LRI	% Peak area
**1**	α-Gurjunene	1411	0.3 ± 0.1

**2**	β-Caryophyllene	1425	7.9 ± 1.8
**3**	Aromadendrene	1440	0.8 ± 0.2
**4**	α-Humulene	1460	3.4 ± 0.7
**5**	Alloaromadendrene	1467	0.7 ± 0.1
**6**	γ-Muurolene	1482	0.2[a]
**7**	Germacrene D	1487	0.2[a]
**8**	β-Selinene	1490	0.2 ± 0.1
**9**	α-Selinene	1493	1.0 ± 0.3
**10**	β-Bisabolene	1501	0.9 ± 0.2
**11**	α-Patchoulene	1521	0.1[a]
**12**	γ-Cadinene	1525	0.1[a]
**13**	δ-Cadinene	1529	0.3[a]
**14**	δ-Selinene	1543	0.6 ± 0.2
**15**	Selina-3,7-diene	1550	0.6 ± 0.1
**16**	trans-Nerolidol	1568	0.5 ± 0.1
**17**	Caryophyllene-oxide	1593	7.8 ± 1.5
**18**	Humulene-1,2-epoxide	1619	2.4 ± 0.5
**19**	Caryophylla-4(12),8(13)-dien-5-ol	1628	0.5[a]

**20**	Cubenol	1641	1.2 ± 0.3
**21**	(Z)-14-Hydroxycaryophyllene	1668	1.3 ± 0.2
**22**	epi-a-Bisabol	1681	1.3 ± 0.3
**23**	β-Bisabolol	2041	0.4 ± 0.1
**24**	Cannabidiol (CBD)	2646	53.2 ± 7.8
**25**	Cannabigerol (CBG)	2838	2.5 ± 0.5
	Total area		88.2 ± 1.1
**[a]SD < 0.05**			

Supplementary Figure 1: Analysis of the CB2r/CB1r mRNA ratio in BV-2 microglia cells analyzed by means of RT-PCR. CB2r mRNA expression (Mean Ct±SD, CBr1: 33.94±1.47, CBr2: 21.33±0.49) in 100 ng of BV-2 CTRL cDNA.

Supplementary Figure 2: Cell viability of BV-2 cells was tested by CCK-8 assay after a 24 h treatment at the concentrations 0.01-0.1-1-10 µg/mL of CSE (**a**), CBD (**b**), and CAR (**c**) respectively. Each column represents mean ± S.E.M. Data were analyzed by one-way ANOVA: ***p<0.001, *p<0.05 vs CTRL group; (n=6). (**d**) BV-2 cell viability was tested by MTT assay following a 24-hour cotreatment of LPS (250 ng/mL) with CSE, CBD, and CAR (1 µg/mL) respectively. Each column represents mean ± S.E.M. Data were analyzed by one-way ANOVA: (n.s.) no significant differences were observed vs CTRL group; (n=6).

Supplementary Figure 3: RT-qPCR analysis of PPARγ mRNA levels in BV-2 cells following the CSE, CBD and CAR (1 µg/mL) treatments in unstimulated and LPS-stimulated BV-2 cells (250 ng/mL). Each column represents mean ± S.E.M. Data were analyzed by one-way ANOVA followed by Tukey: *p<0.05 vs CTRL group (n=5).

Suppl. Figure 1

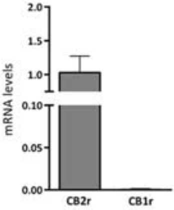

Suppl. Figure 2

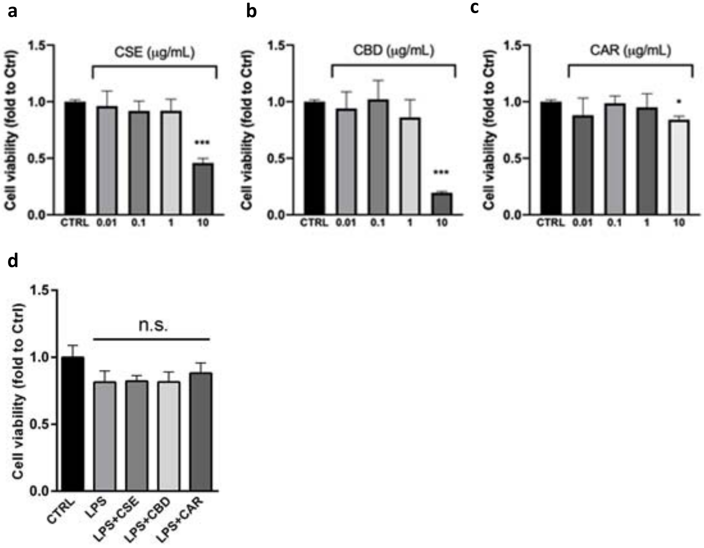

Suppl. Figure 3

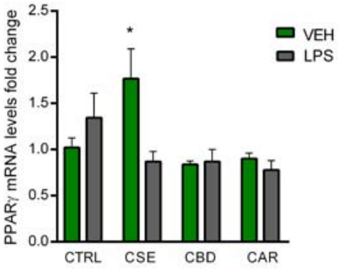

Supplement: Supplementary file 1 — Figure S1. Analysis of the CB2r/CB1r mRNA ratio in BV‐2 microglia cells analyzed by means of RT‐qPCR. CB2r mRNA expression (Mean Ct±SD, CBr1: 33.94±1.47, CBr2: 21.33±0.49) in 100 ng of BV‐2 CTRL cDNA. Figure S2. Cell viability of BV‐2 cells was tested by CCK‐8 assay after a 24 h treatment at the concentrations 0.01‐0.1‐1‐10 μg/mL of CSE (a), CBD (b), and CAR (c) respectively. Each column represents mean ± S.E.M. Data were analyzed by one‐way ANOVA: ***p<0.001, *p<0.05 vs CTRL group; (n = 6). (d) BV‐2 cell viability was tested by MTT assay following a 24‐hr cotreatment of LPS (250 ng/mL) with CSE, CBD, and CAR (1 μg/mL) respectively. Each column represents mean ± S.E.M. Data were analyzed by one‐way ANOVA: (n.s.) no significant differences were observed vs CTRL group; (n = 6). Figure S3. RT‐qPCR analysis of PPARγ mRNA levels in BV‐2 cells following the CSE, CBD and CAR (1 μg/mL) treatments in unstimulated and LPS‐stimulated BV‐2 cells (250 ng/mL). Each column represents mean ± S.E.M. Data were analyzed by one‐way ANOVA followed by Tukey: *p<0.05 vs CTRL group (n = 5). [file PTR-36-2246-s002.pdf]
